# The Effect of Antioxidant Supplementation in Patients with Tinnitus and Normal Hearing or Hearing Loss: A Randomized, Double-Blind, Placebo Controlled Trial

**DOI:** 10.3390/nu11123037

**Published:** 2019-12-12

**Authors:** Anna I. Petridou, Eleftheria T. Zagora, Petros Petridis, George S. Korres, Maria Gazouli, Ioannis Xenelis, Efthymios Kyrodimos, Georgia Kontothanasi, Andriana C. Kaliora

**Affiliations:** 1 1st ENT Department, School of Medicine, National and Kapodistrian University of Athens, Hippokration Hospital, 11527 Athens, Greece; anna.petridou@outlook.com (A.I.P.); cxeneli@yahoo.com (I.X.); timkirodimos@hotmail.com (E.K.); 2 ENT Department, General Hospital of Nikaia “Agios Panteleimon”, Nikaia, 18454 Athens, Greece; zagoraeleftheria@gmail.com (E.T.Z.); georgia.kontothanasi@gmail.com (G.K.); 3 ENT Department, St. Johannes Hospital, 44137 Dortmund, Germany; petridispppeter@hotmail.com; 4 2nd ENT Department, School of Medicine, National and Kapodistrian University of Athens, Attikon Hospital, 12462 Chaidari, Greece; gfkorres@gmail.com; 5 Department of Biology, School of Medicine, National and Kapodistrian University of Athens, 11527 Athens, Greece; maria.gazouli@gmail.com; 6 Department of Dietetics and Nutritional Science, School of Health Science and Education, Harokopio University, 17671 Athens, Greece

**Keywords:** tinnitus, antioxidant supplementation, oxidative stress, a-lipoic acid, multi-vitamin supplement

## Abstract

Tinnitus is the perception of sound in the absence of any external stimulus. Oxidative stress is possibly involved in its pathogenesis and a variety of antioxidant compounds have been studied as potential treatment approaches. The objective of the present study was to assess the effects of antioxidant supplementation in tinnitus patients. This is a randomized, double-blind, placebo-controlled clinical trial. Patients (*N* = 70) were randomly allocated to antioxidant supplementation (*N* = 35) or to placebo (*N* = 35) for a total of 3 months. Demographic, anthropometric, clinical, and nutritional data were collected. Serum total antioxidant capacity (TAC), oxidized LDL (oxLDL), and superoxide dismutase (SOD), tinnitus loudness, frequency, and minimum masking level (MML), and scores in Tinnitus Handicap Inventory questionnaire (THI), Tinnitus Functional Index (TFI), and Visual Analogue Scale (VAS) were evaluated at baseline and follow-up. Tinnitus loudness and MML significantly decreased from baseline to post measure (*p* < 0.001) only in the antioxidant group, the overall change being significantly different between the two groups post-intervention (*p* < 0.001). THI and VAS decreased only in the antioxidant group. Differences in changes in serum TAC, SOD, and oxLDL post-intervention were insignificant. In conclusion, antioxidant therapy seems to reduce the subjective discomfort and tinnitus intensity in tinnitus patients.

## 1. Introduction

Tinnitus is the perception of sound in the absence of any external stimulus [1]. Its prevalence is estimated at approximately 10%–15% of the adult population and its impact varies from a mild perception to a severe disturbance in everyday life [2]. Causes of tinnitus remain unknown and the pathological mechanisms are not fully understood. Currently, no drug treatment is effective, whereas the treatment approaches described in literature provide different results [2].

Oxidative stress has been proposed to play a critical role in the pathogenesis of tinnitus, since it could lead to cellular changes in hair cells, hair cell apoptosis, cochlear degeneration, changes in supporting structures and stria vascularis, changes in nerve fibers of the acoustic nerve, irregular neural activity in the auditory pathway and dysfunction of the central cortex [3,4,5]. Hair cell apoptotic pathways linked to oxidative stress have been studied in animal models in conditions of aging, noise trauma and ototoxicity [3]. Oxidative stress activates mitogen-activated protein kinase/c-Jun N-terminal kinase (MAPK/JNK) pathway, which subsequently leads to the release of cytochrome c from mitochondria [3,6]. Cytochrome c causes mitochondrial membrane damage and activates caspase pathway, promoting apoptosis [3,6]. In organ of Corti cultures, low dose oxidative stress has been shown to induce mitochondrial DNA (mtDNA) deletions in hair cells, which make them more vulnerable to further injury [7]. Moreover, oxidative stress may be linked to endothelial damage within cochlear microcirculation [8].

Higher plasma concentrations of oxidative stress biomarkers and lower antioxidant activity have been reported in tinnitus patients compared with healthy subjects [8,9,10,11,12]. However, research data on the efficacy of antioxidant supplementation in tinnitus are limited and conflicting. Moreover, oxidative stress biomarkers have not been assessed in most studies. Gingko Biloba, a rich source of flavonoids, has been shown to reduce the subjective discomfort and intensity of tinnitus [13,14]. Additionally, preliminary outcomes of a brief report have shown that an 18-week supplementation with antioxidants and phospholipids regulated oxidative stress and reduced the subjective discomfort and intensity of tinnitus [15]. However, other studies failed to show any benefit [16,17].

A great deal of the protective mechanisms of antioxidants against cochlea damage has been identified from animal models For example, alpha-lipoic acid (ALA) has been shown to provide protection from noise-induced hearing loss in animal models [18,19]. Moreover, many experimental studies have proven the protective effect of polyphenols against cisplatin-induced ototoxicity [20,21,22] and cochlear hair cell damage after intense noise exposure [23,24,25]. In human studies, vitamin E has been shown effective in the treatment of sudden idiopathic hearing loss [26] and ALA in the prevention of noise-induced hearing loss [27].

The aim of the study was to explore the efficacy of antioxidant supplementation which provides vitamins, minerals, and phytochemicals combined with ALA on tinnitus parameters and subjective discomfort. A second aim was to assess whether antioxidant administration had an impact on biomarkers of oxidative stress.

## 2. Materials and Methods 

### 2.1. Study Design

The study protocol was reviewed and approved by the Scientific Council of the General Hospital of Athens, “Hippocratio” (11888/24-6-2009) and it was conducted according to the principles of the Declaration of Helsinki of 1975 as revised in 2013.

This was a randomized, double-blind, placebo-controlled, parallel group clinical trial (Appendix A). The trial was registered with ClinicalTrials.gov (Identifier: NCT04105426).

The primary endpoint was change in tinnitus loudness. The secondary endpoints were changes in tinnitus frequency and MML, the impact of tinnitus on daily life, hearing thresholds, and serum oxidative stress biomarkers. Tinnitus patients were enrolled based on certain inclusion and exclusion criteria. Both males and females, aged between 25 and 75 years old, with chronic unilateral or bilateral persistent tinnitus of at least 6 months’ duration, with normal hearing or up to moderate sensorineural hearing loss were included. Tinnitus maskable with noise of at least 5 decibel and a score of minimum 4 in Tinnitus Handicap Inventory (THI) questionnaire were set as inclusion criteria. Patients whose tinnitus resulted from acute acoustic trauma, sudden deafness or traumatic head or neck injury were excluded from trial enrolment. Patients who were taking ototoxic or potentially tinnitus-inducing medication (e.g., aminoglycosides, chemotherapeutics, loop diuretics, high doses of aspirin or quinine) were also excluded. Moreover, patients with Meniere’s disease, otosclerosis and acute or chronic otitis media were excluded. Gastrointestinal disease, active malignant diseases, autoimmune diseases, hemorrhagic diathesis, cardiovascular, renal or hepatic disorders, psychiatric disorders, and unregulated diabetes mellitus, hypertension, or thyroid disease were also set as exclusion criteria. Additionally, alcohol or drug abuse, dietary supplement use, a vegan or macrobiotic diet <2 years prior to screening, pregnancy and lactation were exclusion criteria. Patients who changed their medication, diet or physical activity habits during the trial were also excluded.

One hundred patients registered in medical archives with the symptom of tinnitus were invited for screening. An Ear Nose and Throat (ENT) physician, unrelated to the project, took a complete medical and tinnitus history followed by an ENT review examination including an audiological evaluation. Audiological assessment included conventional pure tone audiometry (PTA), tympanometry and brainstem response (BSR). Moreover, a computer tomography scanning (CT) and magnetic resonance imaging (MRI) were conducted where appropriate, in order to exclude any retrocochlear lesion. Seventy patients were eligible for the study, based on the above criteria. The ENT physician informed the eligible patients regarding the aims, methods, anticipated benefits and potential hazards of the study, and provided them with the information leaflet of the study. Each patient who agreed to take part in the study, signed an informed consent form, a copy of which was given to them.

After the initial screening, participants were randomly allocated to an intervention, either active or placebo. Randomization was conducted by an unrelated to the study person, who prepared a random number list in the computer. Neither the participants nor the investigators were aware of the treatment allocation.

The antioxidant group received one multivitamin-multimineral tablet once a day with their meal and one tablet of alpha-lipoic acid twice a day on an empty stomach, whereas the placebo group received three placebo tablets per day at the same time points. To prevent any acute supplementation effects, participants were asked not to take any tablets on the day of the follow-up measurements. To check upon compliance, each participant was seen monthly by a research coordinator who checked the compliance and tolerance of the supplement. Additionally, blood levels of vitamins and minerals were estimated in all participants before and after the intervention.

The dietary supplements received by the antioxidant group are commercially available. A-lipoic acid supplement contained 300 mg a-lipoic acid per tablet. The ingredients of the multivitamin-multimineral supplement are shown in Appendix B. The nutritional supplementation doses in the multivitamin-multimineral supplement were in line with or above the Recommended Dietary Allowances and Adequate Intakes (RDA) [28] and did not exceed the Tolerable Upper Intake Levels (UL) [29]. These doses are commonly available in commercial multivitamin supplements. Placebo pills were produced by a local manufacturing pharmacy according to good manufacturing practice (GMP) and contained sorbitol. They were manufactured with similar shape and color to the other supplements.

Dietary supplements and placebo pills were packaged in bottles and then in bags of identical appearance and labeled with the participant’s number by an investigator who was not involved in the study. The bags were given to participants by an independent investigator. Instructions for the consumption of pills were included in the bags.

The intervention lasted 3 months. Participants were instructed to keep their usual medical treatment, diet, and exercise habits stable during the intervention. Patients were recruited between January 2019 and March 2019. Follow-up visits were completed on May 2019.

### 2.2. Baseline Assessment

After enrollment, patients underwent a complete baseline assessment which included anthropometric, audiometric, tinnitus psychoacoustic measures, tinnitus discomfort, psychological, physical activity, and dietary assessment as well as blood sample collection.

#### 2.2.1. Audiometric Assessment and Psychoacoustic Measures of Tinnitus

Patients underwent conventional pure tone audiometry and extended high frequency (EHF) audiometry, to determine any hearing loss. Pure tones were delivered to the ear where tinnitus was more intense at the frequencies from 250 to 12,000 Hz. Pure tone audiometric threshold (PTA) is the minimum volume required to hear each tone at the examined frequency. PTA thresholds were then plotted on the audiogram. The degree of hearing loss was determined using the average of values in four consecutive frequencies (500–1000–2000–4000) and was classified as normal hearing, mild hearing loss or moderate hearing loss [30]. High frequency hearing loss was determined based on figures of hearing thresholds at extended high frequencies [31].

Moreover, tinnitus assessment tests were conducted, using psychoacoustic techniques which included frequency pitch and loudness matching, as well as minimum masking level (MML) method [32]. When tinnitus was bilateral, tests were performed to the ear where tinnitus was more intense.

Frequency pitch matching test, which determines the possible frequency of tinnitus, was conducted using the two-forced alternative choice procedure. Patients were given pairs of different tones in the ear without or less intense tinnitus and were asked to choose which tone is closer to the perceived tinnitus. This was continued until a definite match was made [32].

Loudness matching test, which determines the loudness of tinnitus, was conducted using the ascending method. Tones close to or exactly the frequency determined with the pitch matching test were presented to the patients in the ear without or less intense tinnitus. The intensity level started from just below threshold and was increased until there was a match to the perceived tinnitus loudness [32].

The MML method, which determines the least intensity needed to just mask patient’s tinnitus, was conducted using the ascending method. Tones close to or exactly the frequency determined with the pitch matching test were presented to the ear with tinnitus. The intensity level started from below threshold and was increased until the patient stopped to perceive his/her tinnitus [32].

#### 2.2.2. Tinnitus Discomfort Assessment

Patients completed the questionnaires Tinnitus Handicap Inventory (THI), Tinnitus Functional Index (TFI), and the Visual Analogue Scale (VAS), which measure the subjective discomfort a patient experiences because of tinnitus.

THI comprises 25 questions which are divided in functional, emotional, and catastrophic subscales [33]. Total scores of THI range from 0 to 100.

Visual Analogue Scale assessed the annoyance patients experienced because of tinnitus during work, sleep, relaxing, and concentration [34]. Each score of VAS ranged from 0 to 10 and the total score was the mean of the scores.

TFI includes eight subscales which concern different aspects of daily life: Intrusiveness (I), sense of control (SC), cognition (C), sleep (SL), audition (A), relaxation (R), quality of life (Q), and emotions (E) [35]. These sub-scales contribute to a subscale score and to an overall score ranging from 0 to 100.

#### 2.2.3. Anthropometric Assessment

All anthropometric measurements were recorded after a ≥12-h fast. Body weight was measured with light clothing and without shoes using a flat scale (Tanita WB-110MA, Tokyo, Japan) and was recorded to the nearest 0.1 kg. Height was measured on a stadiometer (Seca Model 220, Hamburg, Germany) and was recorded to the nearest 0.1 cm. BMI was calculated as weight (in kg) divided by height^2^ (in m^2^). The waist and hip circumferences were measured using a stretch-resistant tape.

#### 2.2.4. Nutrition and Physical Activity Evaluation

Dietary intake of patients was assessed by a dietitian using the 24-h recall method for 3 nonconsecutive days (2 weekdays and 1 weekend) and a Food Frequency Questionnaire [36]. Nutritional data were then analyzed by Nutritionist Pro nutrient analysis software version 5.2.0 (Axxya Systems, Nutritionist Pro, Stafford, TX, USA). Additionally, adherence to the Mediterranean dietary pattern was assessed by the MedDietScore, resulting to a score ranging from 0 to 55 [37]. Physical activity was assessed by a self-administered long form of the International Physical Activity Questionnaire (IPAQ) [38].

#### 2.2.5. Psychological Assessment

The Center for Epidemiologic Studies-Depression (CES-D) and Hospital Anxiety and Depression (HADS) scales were used to assess the psychological situation of patients. Both scales are self-administered and validated in Greek language [39,40].

#### 2.2.6. Blood Sample Collection and Analyses

Standard blood sampling (20 mL) was performed through a catheter in an antecubital vein after a 12 h overnight fast. Freshly drawn blood samples were used for routine biochemical profiles. Serum and plasma were isolated for further processing.

Vitamin D was measured using the Vitamin D Elisa Kit (Vitamin D Elisa Kit, Cayman Chemical Company, Ann Arbor, MI, USA). Folate was measured using a competitive immunoassay according to the instructions of manufacturers (ADVIA Centaur Folate assay, Siemens Healthcare Diagnostics, NY, USA). Iron was determined using the ADVIA Chemistry Iron_2 method (Siemens Healthcare Diagnostics, NY, USA) [41]. Magnesium was measured using the ADVIA Chemistry Magnesium method which is based on the modified xylidyl blue reaction (Siemens Healthcare Diagnostics, Tarrytown, NY, USA) [42]. Vitamin B12 was determined using a competitive immunoassay according to the instructions of manufacturers (ADVIA Centaur VB12 assay, Siemens Healthcare Diagnostics, Tarrytown, NY, USA). Vitamins A, E, C, B1, B2, and B6 were measured applying high performance liquid chromatography (HPLC) according to the instructions of the manufacturer (ClinRep, HPLC kit, Recipe Chemicals, München, Germany). Zinc was determined using Colorimetric method 5-Br-PAPS-Zinc complex with deproteinization according to the instructions of manufacturers (Wako Zinc Test, FUJIFILM Wako Chemicals Europe, Neuss, Germany). Selenium was measured using a methylene blue kinetic catalytic spectrophotometric method (Sigma-Aldrich Chemie GmbH, Taufkirchen, Germany).

The rest of the samples were stored at −80 °C for subsequent analyses.

### 2.3. Follow-Up Assessment

Compliance and any side effects were checked with a weekly telephone contact. Adherence to supplementation was assessed by counting the remaining pills in the package of each participant at the end of the intervention. At the end of the intervention, all baseline assessments were repeated apart from the psychological and physical activity assessment.

### 2.4. Oxidative Stress Biomarkers

Analyses of oxidative stress and antioxidant capacity biomarkers were performed in the Lab of Biology in Medical School of National and Kapodistrian University of Athens.

Serum total antioxidant capacity (TAC) was measured using the Trolox Equivalent Antioxidant Capacity (TEAC) method according to the instructions of the manufacturer (Cayman Antioxidant Assay Kit, Cayman Chemical Company, Ann Arbor, MI, USA). Serum Superoxide Dismutase (SOD) activity was assessed by measuring the dismutation of superoxide radicals generated by xanthine oxidase and hypoxanthine (Cayman Superoxide Dismutase kit, Cayman Chemical Company, Ann Arbor, MI, USA). Oxidized low-density lipoprotein LDL (oxLDL) was measured by a sandwich enzyme-linked immune-sorbent assay (Human OxLDL ELISA kit, Wuhan Fine Biological Technology, Wuhan, China). Samples and standards were run in duplicate. A Biotek PowerWave XS2 ELISA reader (BioTek Instruments, Winooski, VT, USA) was used for all measurements and analysis.

### 2.5. Sample Size Determination

Power analysis methodology represents a design, with two levels of the between-subject factor of two study groups and two levels of the within-subjects factor of time. A repeated measures ANOVA power analysis was conducted. The effect size for this calculation used the ratio of the standard deviation of the effects for a particular factor or interaction and the standard deviation of within-subject effects. The power analysis was conducted for a single, two-group between-subjects factor, and a single within-subjects factor assessed over two time points. For this design, 68 participants (34 per group) achieves a power of 0.95 for the within-subjects main effect at an effect size of 0.22; and a power of 0.95 for the interaction effect at an effect size of 0.25.

### 2.6. Statistical Analysis

Continuous variables are presented with mean and standard deviation (SD) and/or with median and interquartile range (IQR). Quantitative variables are presented with absolute and relative frequencies. For the comparison of proportions, chi-square and Fisher’s exact tests were used. For the comparison of study variables between the placebo and antioxidant group the Student’s *t*-test was computed. Differences in changes of tinnitus parameters, antioxidant parameters, minerals, and vitamins during the follow up period between the two study groups were evaluated using repeated measurements analysis of variance (ANOVA). Variables that had skewed distribution were log-transformed for the analysis of variance. All *p*-values reported are two-tailed. Statistical significance was set at 0.05 and analyses were conducted using SPSS statistical software (version 22.0) (IBM, Armonk, NY, USA).

## 3. Results

Seventy patients with tinnitus met the criteria for recruitment. Out of the 70 patients, 35 were randomized to the antioxidant group and 35 to the placebo. One patient from the antioxidant group and 2 patients from the placebo group discontinued the intervention due to an unscheduled surgery. Moreover, four patients from the placebo group were lost and unable to contact during the follow-up. Sample consisted of 63 patients (29 in the placebo group and 34 in the antioxidant group). No adverse events were mentioned in either of the two groups. Patient compliance was good. Average missed tablets (days) in antioxidant and placebo group were four and five, respectively.

Demographic, clinical, biochemical, and anthropometric characteristics for both groups are presented in Supplementary Material, Table S1. The mean age was 59.2 years (SD = 13.5 years) for the placebo group and 56.5 years (SD = 12.4 years) for the antioxidant group (*p* = 0.416). Both groups were similar in terms of sex, education, marital status, and smoking habits. Moreover, there were no differences in BMI, waist and hip circumferences and biochemical blood profile. Scores on HADS and CES-D scales along with physical activity levels were also similar for the placebo and antioxidant group (Supplementary Material, Table S1). Table 1 presents the descriptive characteristics of tinnitus and classification of hearing. Tinnitus duration and severity, family history of tinnitus, and hearing loss, as well as the number of previous therapies, the age of tinnitus onset and the presence of normal hearing and hearing loss were similar in the placebo and antioxidant group.

Additionally, the two groups were similar as far as the dietary habits are concerned. There were no differences in baseline total energy and macronutrient intake, as well as adherence to the Mediterranean diet assessed by MedDietScore and the frequency of consumption of herbal beverages, chocolate, coffee, and wine as presented in Supplementary Material, Table S2.

The anthropometric and biochemical parameters before and after the intervention are presented in Supplementary Material, Tables S3 and S4. BMI, waist and hip circumferences remained unchanged in both groups after intervention. Moreover, biochemical parameters did not change, apart from LDL, which increased significantly in the antioxidant group. No changes were also reported for the total energy and macro- and micronutrient dietary intake after intervention for both groups (Supplementary Material, Table S5).

The changes for tinnitus loudness, tinnitus frequency and Minimum Masking Level (MML) for both groups after intervention are presented in Table 2. Loudness and MML significantly decreased from baseline to post measure (*p* < 0.001) only in the antioxidant group and the overall change was different between the two groups as indicated from the significant interaction effect of the analysis (*p* < 0.001). Tinnitus frequency did not significantly change in any of the two groups.

**Figure 1:**
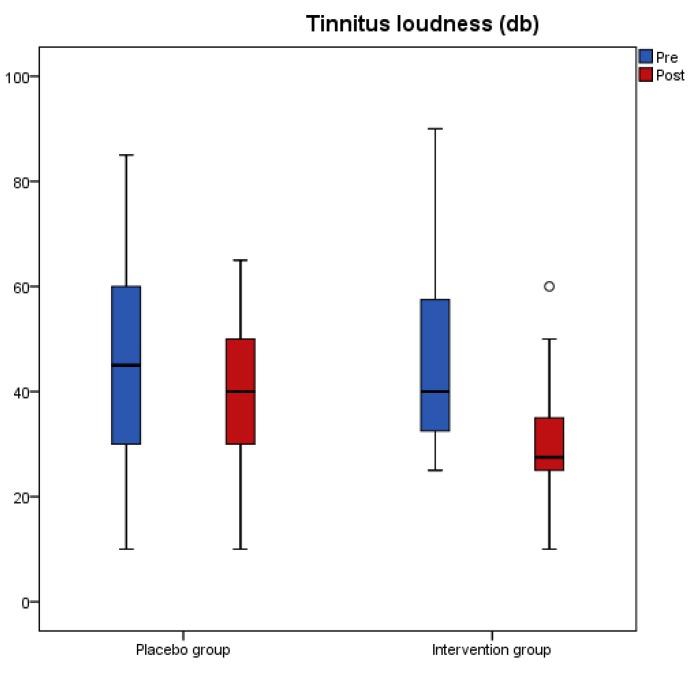
Tinnitus loudness (db) is displayed as box plots for each group at baseline and at follow-up. The line inside the box represents the median and the box portion of the box plot is defined by two lines at the 25th percentile and 75th percentile. Circles denote outliers.

**Figure 2:**
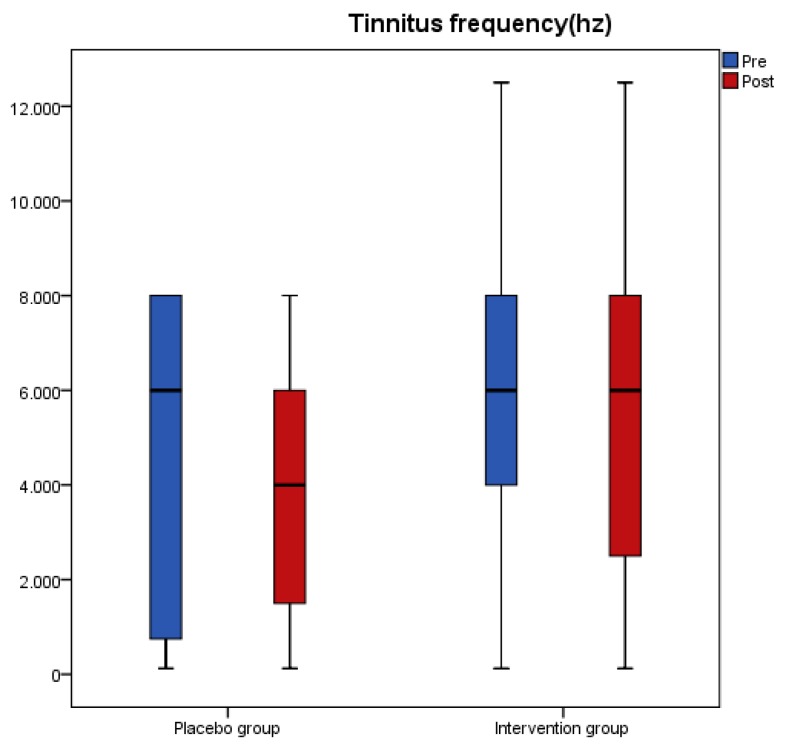
Tinnitus frequency (Hz) is displayed as box plots for each group at baseline and at follow-up. The line inside the box represents the median and the box portion of the box plot is defined by two lines at the 25th percentile and 75th percentile.

**Figure 3:**
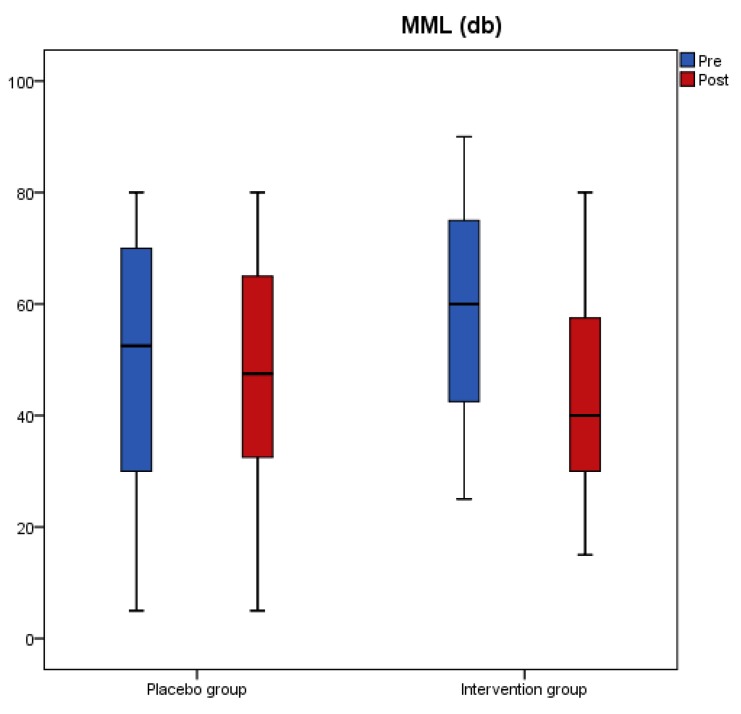
MML (db) is displayed as box plots for each group at baseline and at follow-up. The line inside the box represents the median and the box portion of the box plot is defined by two lines at the 25th percentile and 75th percentile.

**Figure 4:**
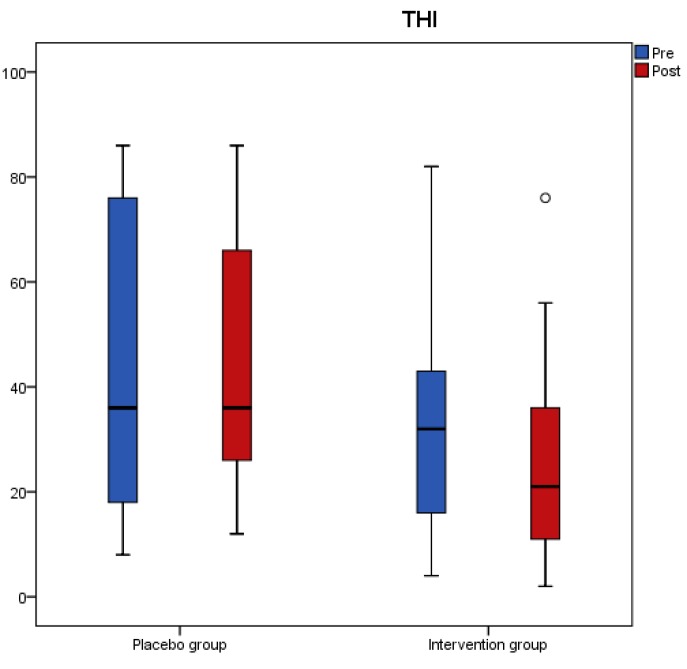
THI is displayed as box plots for each group at baseline and at follow-up. The line inside the box represents the median and the box portion of the box plot is defined by two lines at the 25th percentile and 75th percentile. Circles denote outliers.

Results from the tinnitus questionnaires before and after the intervention revealed that scores of THI, VAS, TFI-Relaxation (TFI-R), and TFI-Emotions (TFI-E) had a significant reduction in the antioxidant group, while no change was recorded in the placebo group (Table 3). A significant interaction effect of group with time indicated a significant treatment difference for THI, TFI-R, and TFI-E (Table 3). Figure 1, Figure 2, Figure 3 and Figure 4 present the median values for tinnitus loudness, tinnitus frequency, MML, and THI accordingly, displayed as box plots for each group.

To assess if tinnitus duration had any effect on the outcomes, placebo and antioxidant groups were divided in two subgroups according to tinnitus duration. There were no differences in tinnitus loudness, tinnitus frequency, MML and THI between patients with tinnitus duration lower and higher than 10 years at baseline and at follow-up in placebo (Table 4) and antioxidant group (Table 5).

As far as serum concentrations of biomarkers of oxidative stress are concerned (Table 6), serum TAC was decreased significantly in both groups, while SOD and oxLDL did not change in either of the two groups. Differences between the groups in changes in serum TAC, SOD and oxLDL postintervention were insignificant.

**Figure 5:**
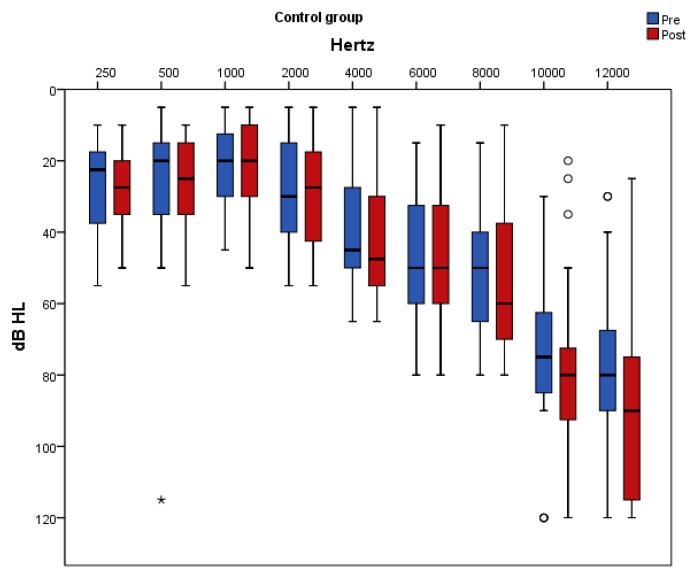
Pure-tone thresholds in the frequency range from 250 to 12,000 Hz (dB HL) are displayed as box plots for each frequency in the placebo group at baseline and at follow-up. The line inside the box represents the median and the box portion of the box plot is defined by two lines at the 25th percentile and 75th percentile. Circles denote outliers and asterisks denote extreme values.

**Figure 6:**
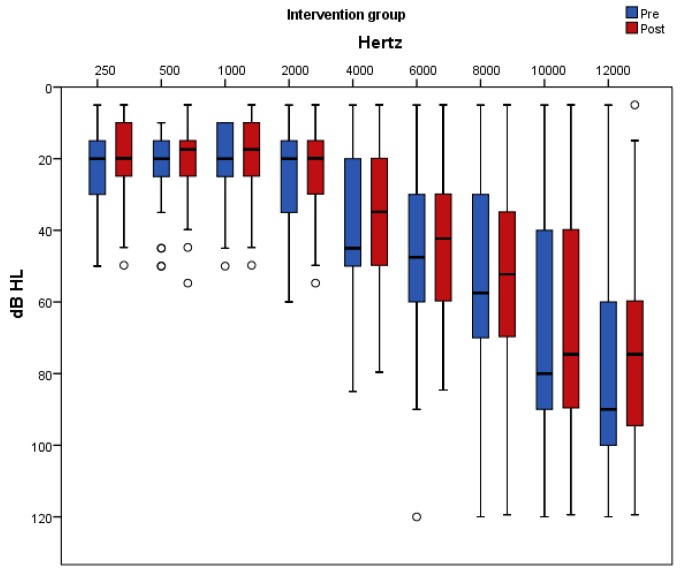
Pure-tone thresholds in the frequency range from 250 to 12,000 Hz (dB HL) are displayed as box plots for each frequency in the antioxidant group at baseline and at follow-up. The line inside the box represents the median and the box portion of the box plot is defined by two lines at the 25th percentile and 75th percentile. Circles denote outliers.

Figure 5 and Figure 6 present pure-tone thresholds in the frequency range from 250 to 12,000 Hz (dB HL) as box plots for each frequency at baseline and at follow-up in the placebo and antioxidant group accordingly. The degree of change in the PTA thresholds at the frequencies of 250 Hz, 2000 Hz, 4000 Hz, 10,000 Hz, and 12,000 Hz differed significantly between the two groups. Specifically, at the frequencies of 250 Hz, 500 Hz, 1000 Hz, 2000 Hz, and 6000 Hz there was a significant decrease in the auditory threshold only in the antioxidant group whereas in the placebo group there was no significant change post intervention. At the frequency of 10,000 Hz there was a significant increase only in the placebo group while in the antioxidant group there was no significant change after the intervention. At the frequencies of 4000 Hz and 12,000 Hz there was a significant decrease in the antioxidant group and a significant increase in the placebo group.

As a measure of compliance, blood status of vitamins and minerals was evaluated and results are presented in Table 7. Vitamin D, folate, vitamin B2, vitamin B1, and vitamin B6 levels increased significantly in the antioxidant group postintervention, whereas iron, magnesium, zinc, selenium, vitamin B12, vitamin E, and vitamin C levels remained unchanged. The estimated treatment difference was significant for folic acid as indicated from the interaction effect of the analysis (*p* = 0.049). In the placebo group, vitamin and mineral levels did not change post-intervention.

## 4. Discussion

Herein, the efficacy of antioxidant supplementation with vitamins, minerals and phytochemicals combined with ALA on tinnitus parameters and subjective discomfort has been investigated. Furthermore, the effect of antioxidant supplementation on biomarkers of oxidative stress has been assessed.

Given that tinnitus is a symptom that has multiple dimensions, psychoacoustic measures of tinnitus (loudness, frequency, and MML) together with questionnaires assessing the tinnitus subjective discomfort (THI, TFI, and VAS-annoyance scale) were applied in the present study in order to assess the efficacy of antioxidant supplementation. This was done in accordance with the Consensus for tinnitus patient assessment and treatment outcome measurement [43]. Separate assessment of psychoacoustic features of the tinnitus and tinnitus related distress in everyday life is highly recommended, since they have a weak correlation with each other [44,45]. Our findings showed that tinnitus loudness, MML and scores in THI, VAS, TFI-relaxation, and TFI-emotions decreased significantly only in the antioxidant group, with the overall changes being significantly different between the two groups post-intervention. This means that antioxidant supplementation was effective in improving tinnitus’ sensory aspects (tinnitus loudness and MML) and in alleviating patients from tinnitus-related distress compared to placebo. The mean reduction in the THI score of 6 points in the antioxidant group, is considered a clinically relevant change [46]. In accordance with present findings, a significant reduction in the VAS scale and tinnitus loudness was reported after an 18-week supplementation with a mix of phospholipids and vitamins [15].

At the same time, this study showed that antioxidant supplementation led to significant improvements of hearing thresholds across all frequencies, with the overall changes being significantly different between the two groups at the frequencies of 250 Hz, 2000 Hz, 4000 Hz, 10,000 Hz, and 12,000 Hz. Improved thresholds at frequencies between 250 Hz and 8000 Hz is of high importance, since these are the frequencies used for speech recognition and thus tinnitus patients could benefit from a better understanding of speech and language.

Despite the key role of oxidative stress in the pathogenesis of tinnitus, data on the efficacy of antioxidant supplementation on oxidative stress biomarkers in tinnitus patients are scarce. This study assessed oxidative stress by measuring serum oxLDL. OxLDL, measured in blood using immunological methods (i.e., antibodies), is considered by the European Food Safety Authority (EFSA) as a reliable in vivo marker of oxidative damage with appropriate specificity [47]. Moreover, serum TAC and SOD were measured in order to assess total antioxidant status and endogenous antioxidant activity accordingly. No statistically significant differences were observed in changes in serum TAC, SOD, and oxLDL in the antioxidant group compared to placebo group post-intervention. These results are consistent with other studies which have been done in healthy adults or adults with concomitant diseases (i.e., diabetes, CVD) and have shown that antioxidant supplementation had no effect on ox.LDL [48,49], SOD [50,51,52], or TAC [51,52]. The absence of any effect of antioxidant supplementation on serum ox. LDL and TAC may be due to the low doses of vitamins C (150 mg) and a-tocopherol (150 IU) in the supplement used. As such, the plasma levels of vitamins C and E in the antioxidant group post-intervention remained unchanged. TAC measures total endogenous and food-derived antioxidants and its values are affected by serum concentrations of vitamins E and C [53]. Moreover, it has been shown that a significant reduction in LDL susceptibility to oxidation could be achieved with a minimum dose of 400 IU/d of α-tocopherol supplementation [54].

Antioxidant supplementation studies in tinnitus are limited. Moreover, in most of them a monotherapy treatment approach has been proposed such as zinc [16,55,56], with no benefit in tinnitus. On the contrary, our hypothesis was that an antioxidant combination might be more effective compared with single nutrients, since various antioxidants have a synergistic/complementary activity [57]. A-lipoic acid was used hereby, at a dose of 600 mg daily that has been demonstrated to increase lipoic acid levels in plasma or cells [58,59]. Moreover, the standardized grape seed extract (GSE) contained in the multivitamin-multimineral supplement constitutes a rich source of phenolic compounds including epicatechin, resveratrol and procyanidin oligomers [60]. To the best of our knowledge, this is the first randomized double-blind placebo-controlled study which used a mixture of vitamins, minerals, phytochemicals and ALA in tinnitus.

The beneficial effect observed on tinnitus in this study is most likely attributed to the protective mechanisms of antioxidants contained in the supplements against ROS-induced cochlear hair cell damage. This has been verified from experiments using animal models, in-vitro assays, or auditory cell lines in cases of ototoxicity, age-related or noise-induced hearing loss. A recent study using cochlea explant culture from mice showed that resveratrol, DL-α-lipoic acid and a-tocopherol protected against gentamicin-induced hair cell loss [61]. In animal experiments, vitamins E or C treatment resulted in better auditory sensitivity and less mtDNA deletions with aging [62] and vitamin E or a-lipoic acid attenuated the noise-induced increase of cochlear malondialdehyde [18]. Moreover, a combination of vitamins B1, B2, B6, E, and C was effective in protecting against cisplatin ototoxicity [63] and α-lipoic acid inhibited the kanamycin-induced high expression of phosphorylated p38 and phosphorylated JNK, which mediate cochlear hair cell apoptosis [64]. In studies using auditory cell lines, treatment with D-α-tocopherol or epicatechin reduced the cisplatin-induced increase of ROS and decreased cellular necrosis and apoptosis [65,66].

Apart from their antioxidant mediated effects, compounds in the supplements used may have acted via additional mechanisms. ALA has been widely researched as a neuroprotectant [67] and vitamin B complex play a key role in the functioning of nerve tissue, cellular metabolism, vascular function, and myelin synthesis [68]. Low levels of Vitamin B12 and folate have been associated with decreased endocochlear potential due to the destruction of the microvasculature of the stria vascularis [69]. Moreover, a diet deficient in folate in animals may lead to impaired cochlear homocysteine metabolism and oxidative stress [70].

Despite the interesting results of this study and the advantage of being randomized and double-blinded, it has some limitations, including the enrollment of patients with high levels of oxidative stress (e.g., smokers, diabetics, elderly), the presence of compounds other than antioxidants in the supplement used and the heterogeneity of participants as far as tinnitus duration and severity, hearing loss, family history and age of tinnitus onset are concerned, shown to influence the response to treatment [71,72]. However, these limitations are compensated by the tight control of participants to ensure their compliance with the protocol, as well as the long follow-up and the adequate sample size of the study.

## 5. Conclusions

Results of this study showed that antioxidant supplementation with vitamins, minerals, phytochemicals and ALA could exhibit favorable effects in tinnitus by reducing the subjective discomfort and tinnitus intensity. However, the effect of this antioxidant supplementation in oxidative stress biomarkers in tinnitus patients needs further investigation.

## Figures and Tables

**Table 1:** Descriptive characteristics of tinnitus and classification of hearing. The results are given as *N* (%) of the total number.

	Placebo Group	Antioxidant Group	*p*
	*N* (%)	*N* (%)	
Tinnitus duration (years)			
<1	3 (10.3)	3 (8.8)	0.547 ^++^
1–2	4 (13.8)	9 (26.5)	
2–3	3 (10.3)	5 (14.7)	
3–5	9 (31)	6 (17.6)	
5–10	2 (6.9)	5 (14.7)	
>10	8 (27.6)	6 (17.6)	
Tinnitus onset			
Gradually	14 (48.3)	15 (44.1)	0.741 ^+^
Abruptly	15 (51.7)	19 (55.9)	
Perceived all day	22 (75.9)	24 (70.6)	0.638 ^+^
Site of the tinnitus			
Right ear	3 (10.3)	3 (8.8)	0.603 ^++^
Left ear	12 (41.4)	9 (26.5)	
Both ears	13 (44.8)	20 (58.8)	
Inside the head	1 (3.4)	2 (5.9)	
History of exposure to noise	12 (41.4)	18 (52.9)	0.360 ^+^
Stable loudness all the days	14 (48.3)	17 (50)	0.891 ^+^
Description of tinnitus			
Whistle	14 (48.3)	18 (52.9)	0.493 ^++^
Clatter	0 (0)	2 (5.9)	
Cicadas’ noise	8 (27.6)	7 (20.6)	
Blow	2 (6.9)	3 (8.8)	
Buzzing	3 (10.3)	2 (5.9)	
Other	2 (6.9)	0 (0)	
Bees’ noise	0 (0)	2 (5.9)	
Previous therapies			
0	11 (37.9)	21 (61.8)	0.063 ^++^
1	15 (51.7)	8 (23.5)	
2	1 (3.4)	4 (11.8)	
3	2 (6.9)	1 (2.9)	
Tinnitus severity			
THI, mean (SD)	40.6 (27.7)	31.6 (19.3)	0.139 ^‡^
Tinnitus loudness (db), mean (SD)	47.1 (20.5)	45 (15.3)	0.649 ^‡^
Tinnitus frequency (Hz), mean (SD)	4431 (3078.3)	5562.5 (3027.7)	0.147 ^‡^
MML(db), mean (SD)	49.3 (22.5)	57.9 (18.9)	0.110 ^‡^
Age of tinnitus onset (years), mean (SD)	54 (14.3)	48.2 (15.7)	0.138 ^‡^
Family history of hearing loss	7 (25)	5 (14.7)	0.307 ^+^
Family history of tinnitus	5 (17.9)	6 (17.6)	1.000 ^++^
Classification of hearing			
Normal hearing in conventional and EHF audiometry	10 (35.7)	16 (47.1)	
Mild sensorineural hearing loss in conventional audiometry	14 (50)	13 (38.2)	0.651 ^++^
Moderate sensorineural hearing loss in conventional audiometry	4 (14.3)	5 (14.7)	
High-frequency sensorineural hearing loss in EHF audiometry	11 (37.9)	9 (26.5)	

^+^ Pearson’s chi-square test; ^++^ Fisher’s exact test; ^‡^ Student’s *t*-test. THI: Tinnitus Handicap Inventory questionnaire; EHF: extended high frequency.

**Table 2:** Tinnitus loudness, frequency, and minimum masking level (MML) at baseline and at follow-up.

		Pre		Post		Change	*p* ^1^	*p* ^2^
		Mean (SD)	Median (IQR)	Mean (SD)	Median (IQR)	Mean (SD)		
Tinnitus loudness (db)	Placebo	47.1 (20.5)	50 (30; 60)	40.4 (15.5)	40 (30; 50)	−6.7 (8.8)	0.168	<0.001
	Antioxidant	45 (15.3)	40 (35; 55)	30.8 (11.2)	27.5 (25; 35)	−14.2 (12.7)	<0.001	
Tinnitus frequency(hz)	Placebo	4431 (3078.3)	6000 (1000; 8000)	4240 (2932.6)	4000 (1500; 6000)	−191 (1401.3)	0.303	0.082
	Antioxidant	5562.5 (3027.7)	6000 (4000; 8000)	5222.7 (2982.1)	6000 (2500; 8000)	−339.8(1565.8)	0.216	
MML (db)	Placebo	49.3 (22.5)	52.5 (30; 70)	47.7 (21)	47.5 (32.5; 65)	−1.6 (7.4)	0.989	<0.001
	Antioxidant	57.9 (18.9)	60 (40; 75)	43.4 (16.5)	40 (30; 60)	−14.5 (14.3)	<0.001	

^1^*p*-value for the time effect (using logarithmic transformations); ^2^*p*-value from repeated measurements ANOVA. The effects reported include differences between the groups in the degree of change (using logarithmic transformations).

**Table 3:** Tinnitus questionnaires’ scores at baseline and at follow-up.

		Pre		Post		Change	*p* ^1^	*p* ^2^
		Mean (SD)	Median (IQR)	Mean (SD)	Median (IQR)	Mean (SD)		
THI	Placebo	40.6 (27.7)	30 (16; 74)	42.8 (24.5)	36 (26; 66)	2.2 (11.5)	0.607	0.015
	Antioxidant	31.6 (19.3)	33 (16; 44)	25.5 (18)	21 (11; 36)	−6.1 (11.7)	0.002	
VAS	Placebo	5.13 (2.81)	5 (3.75; 7.25)	4.86 (2.53)	4.5 (3.25; 6.6)	−0.3(2.11)	0.767	0.147
	Antioxidant	3.66 (2.49)	3.38 (1.25;5.75)	2.88 (2.37)	2.38 (0.75; 4.63)	−0.78 (1.4)	0.013	
TFI	Placebo	37.9 (19.7)	32 (22.8; 52)	41 (22.3)	32.8 (27.2; 57.6)	3.1 (10.3)	0.950	0.410
	Antioxidant	30.4 (20)	29.2 (11.4; 47)	28.6 (20.5)	21.2 (13.6; 49.6)	−1.8 (16.1)	0.129	
TFI-I	Placebo	56.9 (26.4)	60 (31.7; 75)	62.7 (26.6)	70 (45; 83.3)	5.7 (17.2)	0.518	0.666
	Antioxidant	48.3 (27.3)	45 (23.3; 71.7)	48.3 (27.8)	43.3 (20; 76.7)	0.1 (20.5)	0.873	
TFI-SC	Placebo	61.9 (27)	56.7 (48.3;86.7)	65.9 (21.8)	60 (56.7; 76.7)	4 (10.9)	0.964	0.376
	Antioxidant	44.2 (29.3)	40 (20; 66.7)	39.9 (27.7)	40 (20; 56.7)	−4.3 (21.3)	0.120	
TFI-C	Placebo	31 (31.4)	30 (0; 45)	22.1 (26.4)	13.3 (0; 30)	−8.9 (29.2)	0.294	0.939
	Antioxidant	31.6 (28.1)	23.3 (8.3; 51.7)	27.7 (26.9)	13.3 (3.3; 60)	−3.8 (23.4)	0.187	
TFI-SL	Placebo	33.6 (32.7)	26.7 (3.3; 61.7)	36.9 (32.7)	23.3 (10; 66.7)	3.3 (6.9)	0.720	0.236
	Antioxidant	23.1 (26.9)	13.3 (0; 45)	23 (30.3)	10 (0; 46)	−0.1 (24.6)	0.128	
TFI-A	Placebo	31.2 (32.4)	23.3 (0; 56.7)	23.5 (31.9)	6.7 (0; 50)	−7.8 (11.7)	0.612	0.836
	Antioxidant	24.2 (25.2)	13.3 (0; 40)	23.9 (23.7)	16.7 (3.3; 36.7)	−0.3 (18)	0.726	
TFI-R	Placebo	49.3 (31.8)	55 (18.3; 73.3)	60.8 (22.7)	66.7 (43.3; 76.7)	11.5(19.1)	0.189	0.037
	Antioxidant	34.7 (29.6)	25 (8.3; 65)	26.5 (26.2)	13.3 (3.3; 50)	−8.2 (22.5)	0.044	
TFI-Q	Placebo	27.2 (29)	20 (0; 50)	24.9 (29.3)	25 (0; 37.5)	−2.4 (11)	0.842	0.897
	Antioxidant	14.3 (16.2)	10 (0; 30)	18.1 (21.1)	10 (0; 25)	3.8 (17.7)	0.957	
TFI-E	Placebo	41.2 (36.7)	30 (10; 68.3)	41 (36.3)	30 (10; 73.3)	−0.2 (10.8)	0.789	0.042
	Antioxidant	29.2 (26)	20 (6.7; 50)	23.3 (22.6)	20 (6.7; 40)	−5.8 (18.1)	0.006	

^1^*p*-value for the time effect (using logarithmic transformations); ^2^*p*-value from repeated measurements ANOVA. The effects reported include differences between the groups in the degree of change (using logarithmic transformations). THI: Tinnitus Handicap Inventory, VAS: Visual Analogue Scale; TFI: Tinnitus Functional Index.

**Table 4:** Tinnitus loudness, frequency, MML, and THI in patients with tinnitus duration of <10 years and >10 years at baseline and at follow-up in placebo group.

	Tinnitus Duration				P Mann-Whitney Test
	<10 Years		>10 Years		
	Mean (SD)	Median (IQR)	Mean (SD)	Median (IQR)	
Tinnitus loudness (db) (pre)	46.9 (22.8)	50 (30; 60)	47.5 (13.9)	47.5 (35; 60)	0.769
Tinnitus loudness (db) (post)	39.7 (16.3)	42.5 (25; 50)	42.1 (14.1)	35 (30; 60)	0.784
Tinnitus frequency (Hz) (pre)	4011.9 (3230)	4000 (750; 8000)	5531.3 (2487.2)	6000 (5000; 7000)	0.345
Tinnitus frequency (Hz) (post)	3875 (3082.2)	4000 (1000; 8000)	5178.6 (2461)	6000 (4000; 6000)	0.373
MML (db) (pre)	47.5 (24.3)	45 (30; 70)	53.8 (17.7)	55 (42.5; 65)	0.557
MML (db) (post)	45.3 (22.7)	45 (25; 65)	53.6 (16.3)	60 (35; 65)	0.426
THI (pre)	42 (30.5)	30 (14; 76)	36.8 (19.3)	31 (22; 48)	0.961
THI (post)	46.1 (27.5)	46 (16; 76)	34.3 (11.7)	30 (26; 50)	0.467

**Table 5:** Tinnitus loudness, frequency, MML and scores in THI in patients with tinnitus duration of <10 years and >10 years at baseline and at follow-up in antioxidant group.

	Tinnitus Duration				P Mann-Whitney Test
	<10 Years		>10 Years		
	Mean (SD)	Median (IQR)	Mean (SD)	Median (IQR)	
Tinnitus loudness (db) (pre)	43 (13.7)	40 (30; 52.5)	54.2 (20.1)	45 (40; 65)	0.199
Tinnitus loudness (db) (post)	30.6 (11.5)	27.5 (25; 35)	31.7 (10.8)	30 (25; 35)	0.825
Tinnitus frequency (Hz) (pre)	5183 (3187.5)	4500 (2000; 8000)	7333.3 (1032.8)	8000 (6000; 8000)	0.080
Tinnitus frequency (Hz) (post)	4889.4 (3142.9)	5000 (2000; 8000)	6666.7 (1633)	7000 (6000; 8000)	0.152
MML (db) (pre)	56.9 (18.7)	55 (40; 75)	62.5 (21.2)	65 (60; 70)	0.574
MML (db) (post)	43.2 (16.4)	40 (30; 55)	44.2 (18.6)	40 (30; 60)	1.000
THI (pre)	30.4 (17.9)	29 (16; 43)	37.3 (26.3)	37 (20; 44)	0.586
THI (post)	23.5 (15.9)	20 (10; 36)	34 (25.4)	30 (12; 48)	0.371

**Table 6:** Oxidative stress biomarkers at baseline and at follow-up.

		Pre		Post		Change	*p* ^1^	*p* ^2^
		Mean (SD)	Median (IQR)	Mean (SD)	Median (IQR)	Mean (SD)		
serum TAC (m M)	Placebo	5.5 (1.5)	5.2 (4.5; 7)	3.9 (2.9)	2.4 (1.4; 6.6)	−1.7 (3.5)	0.002	0.420
	Antioxidant	6.3 (1.9)	6.1 (5.3; 7.2)	5.1 (2.7)	5.5 (3.5; 7.2)	−1.1 (3.8)	0.019	
SOD (U/mL)	Placebo	3.2 (2)	2.5 (2.2; 2.9)	3.1 (1.8)	2.6 (2.2; 3.2)	−0.1 (1.4)	0.792	0.154
	Antioxidant	4 (2.5)	3.3 (2.6; 4.4)	3 (1.6)	2.6 (2; 3.3)	−1 (2.3)	0.065	
ox.LDL (ng/mL)	Placebo	12.1 (23.2)	7 (2; 10.9)	10.4 (8.5)	9 (5.1; 11.4)	−1.7 (7.9)	0.062	0.232
	Antioxidant	25 (50.7)	7.7 (3.8; 15.8)	18.7 (37.6)	8.8 (7.8; 9.6)	−6.3 (27.9)	0.399	

^1^*p*-value for the time effect; ^2^*p*-value from repeated measurements ANOVA. The effects reported include differences between the groups in the degree of change (using logarithmic transformations).

**Table 7:** Blood status of vitamins and minerals at baseline and at follow-up. Values are expressed as the mean ± SD.

		Pre	Post	Change	*p* ^1^	*p* ^2^
		Mean (SD)	Mean (SD)	Mean (SD)		
Vitamin D (ng/mL)	Placebo Antioxidant	0.32 (0.23) 0.32 (0.22)	0.28 (0.13) 0.4 (0.42)	−0.05 (0.16) 0.08 (0.36)	0.657 0.032	0.079
Magnesium (mg/dL)	Placebo	2.06 (0.11)	2.03 (0.1)	−0.03 (0.16)	0.834	0.355
	Antioxidant	2.01 (0.12)	2.07 (0.16)	0.06 (0.17)	0.066	
Zn (μg/dL)	Placebo	105.8 (14.8)	98.6 (11)	−7.1 (12.7)	0.128	0.148
	Antioxidant	111.6 (15.4)	112 (15.7)	0.5 (23.4)	0.777	
B12 (pg/mL)	Placebo	465.3 (330.6)	358.3 (167.8)	−106.9 (378.2)	0.080	0.095
	Antioxidant	359.8 (130.3)	411.9 (98.5)	52.1 (110.5)	0.657	
Folate (ng/mL)	Placebo	8.16 (4.99)	6.45 (2.14)	−1.71 (1.13)	0.463	0.049
	Antioxidant	8.65 (5.21)	11.31 (5.41)	2.66 (4.29)	0.001	
Fe (μg/dL)	Placebo	128.2 (42.1)	118.5 (62.5)	−9.6 (65.6)	0.963	0.309
	Antioxidant	117.8 (36.2)	104.7 (41)	−13.1 (36)	0.134	
Selenium (μg/L)	Placebo	76.5 (11.8)	68.2 (7)	−8.3 (15.4)	0.080	0.095
	Antioxidant	78.4 (9.5)	80.1 (11.3)	1.7 (13.4)	0.730	
Vitamin E (mg/L)	Placebo	12.09 (0.94)	11.68 (1.04)	−0.41 (0.79)	0.908	0.798
	Antioxidant	11.42 (1.16)	11.92 (1.15)	0.5 (1.56)	0.411	
Vitamin C (μg/L)	Placebo	6.73 (1.65)	7.03 (2.73)	0.31 (3.65)	0.324	0.530
	Antioxidant	7.25 (1.82)	7.73 (2.41)	0.48 (3.22)	0.509	
Vitamin B2 (μg/L)	Placebo	220 (22.4)	223.6 (18)	3.6 (11.1)	0.918	0.326
	Antioxidant	221.7 (25.2)	238.4 (26.6)	16.7 (32.4)	0.039	
Vitamin B1 (μg/L)	Placebo	55 (12.4)	56.8 (14.8)	1.8 (27.2)	0.757	0.468
	Antioxidant	50.4 (11.1)	61.5 (13.5)	11.1 (17.7)	0.023	
Vitamin B6 (μg/L)	Placebo	21.5 (3.6)	22.8 (5.6)	1.3 (6)	0.911	0.463
	Antioxidant	24.2 (17.2)	35.6 (12.9)	11.4 (19.9)	0.050	

^1^*p*-value for the time effect; ^2^*p*-value from repeated measurements ANOVA. The effects reported include differences between the groups in the degree of change (using logarithmic transformations).

## Supplementary Materials

The following are available online at https://www.mdpi.com/2072-6643/11/12/3037/s1, Table S1: Sample characteristics by study group, Table S2: MedDietScore, antioxidant food consumption frequency and macronutrient intake by study group, Table S3: Anthropometrics at baseline and at follow up. Values are expressed as the mean ± SD, Table S4: Biochemical parameters at baseline and at follow up, Table S5: Macro- and micronutrient intake at baseline and at follow-up.

## Appendix B

**Table:**

Composition of the Multivitamin-Multimineral Supplement (1 Table)					
Ingredient	Amount per tablet	%RDA	Ingredient	Amount per tablet	%RDA
Vitamin A (acetate)	781 μg (2600 iu)	98	Magnesium (as oxide)	50 mg	13
Vitamin D_3_	10 μg (400 iu)	200	Zinc (gluconate)	15 mg	150
Vitamin E (dl-alpha tocopherol acetate)	100 mg (150 iu)	833	Copper (gluconate)	1.2 mg	120
Vitamin C (Ascorbic acid)	150 mg	188	Manganese (gluconate)	4 mg	200
Thiamine (Vitamin B1) (mononitrate)	25 mg	2272	Selenium (as L-Selenomethionine and sodium selenite)	100 μg	200
Riboflavin (Vitamin B2)	25 mg	1786	Chromium (picolinate)	200 μg	500
Niacin (Vitamin B3)	25 mg	156	Molybdenum (as molybdate)	500 μg	1000
Pyridoxine (Vitamin B6) (Pyridoxine Hydrochloride)	10 mg	714	Iodine (potassium iodide)	150 μg	100
Folic acid	200 μg	100	Choline (Bitartrate)	25 mg	
Vitamin B12	10 μg	400	Inositol	25 mg	
Biotin	150 μg	300	PABA	25 mg	
Pantothenic acid (Vitamin B5) (calcium pantothenate)	25 mg	417	Grapeseed extract (GSE) (Provided by 1mg of a 500:1 extract)	500 mg	
Calcium (as phosphate)	62 mg	8			
Iron (ferrous fumarate)	14 mg	100			
