# Supplementary material for: The Effect of Antioxidant Supplementation in Patients with Tinnitus and Normal Hearing or Hearing Loss: A Randomized, Double-Blind, Placebo Controlled Trial
Source: Nutrients. 2019 Dec 12;11(12):3037. doi: 10.3390/nu11123037 (PMC6950042; doi:10.3390/nu11123037)
Supplement: Supplementary file 1 [file nutrients-11-03037-s001.pdf]

SUPPLEMENTARY MATERIAL RELATED TO

The Effect of Antioxidant Supplementation in Idiopathic Tinnitus: A randomised, Double-Blind, Placebo Controlled Trial

Anna I. Petridou[1], Eleftheria T. Zagora[2], Petros Petridis[3], George S. Korres[4], Maria Gazouli[5], Ioannis Xenelis[1], Efthymios Kyrodimos[1], Georgia Kontothanasi[2] and Andriana C. Kaliora[6,*]

[1] 1st ENT Department, School of Medicine, National and Kapodistrian University of Athens, Hippokration Hospital, 11527 Athens, Greece; anna.petridou@outlook.com; cxeneli@yahoo.com; timkirodimos@hotmail.com

[2] ENT Department, General Hospital of Nikaia "Agios Panteleimon", 18454 Nikaia, Greece; zagoraeleftheria@gmail.com; georgia.kontothanasi@gmail.com

[3] ENT Department, St. Johannes Hospital, 44137 Dortmund, Germany; petridispppeter@hotmail.com

[4] 2nd ENT Department, School of Medicine, National and Kapodistrian University of Athens, Attikon Hospital, 12462 Chaidari, Greece; gfkorres@gmail.com

[5] Department of Biology, School of Medicine, National and Kapodistrian University of Athens , 11527 Athens, Greece; maria.gazouli@gmail.com

[6] Department of Dietetics and Nutritional Science, School of Health Science and Education, Harokopio University, 17671 Athens, Greece; akaliora@hua.gr; andrianakaliora@gmail.com

* Correspondence: akaliora@hua.gr; andrianakaliora@gmail.com. Tel.: +302109549226

Table S1. Sample characteristics by study group

	Placebo group N=29	Antioxidant group N=34	*P*
	N (%)	N (%)	
**Age** (years), mean (SD)	59.2 (13.5)	56.5 (12.4)	0.416‡
**Sex**			
Men	16 (55.2)	25 (73.5)	0.128+
Women	13 (44.8)	9 (26.5)	
**Marital status**			
Married	18 (62.1)	21 (61.8)	0.182++
Unmarried	5 (17.2)	8 (23.5)	
Widowed	5 (17.2)	1 (2.9)	
Divorced	1 (3.4)	4 (11.8)	
**Education (years)**			
1-9	10 (34.5)	4 (11.8)	0.093+
10-12	10 (34.5)	17 (50)	
>12	9 (31)	13 (38.2)	
**Smoking**			
No	14 (48.3)	14 (41.2)	0.671+
Yes	5 (17.2)	9 (26.5)	
In the past	10 (34.5)	11 (32.4)	
**Passive smoking**	5 (17.2)	9 (26.5)	0.308+
**Medical history**			
Hypercholesterolemia	13 (44.8)	11 (32.4)	0.310+
Diabetes	3 (10.3)	1 (2.9)	0.326++
Hypertension	12 (41.4)	8 (23.5)	0.129+
Hypothyroidism	7 (24.1)	3 (8.8)	0.165++
**Psychological situation**			
HADS-A, mean (SD)	5.9 (4.9)	4.5 (3.3)	0.175‡
HADS-D, mean (SD)	4.1 (3.6)	4 (3.5)	0.965‡
CES-D, mean (SD)	12.4 (9.3)	16.1 (10.5)	0.156‡
**Physical Activity**			
MET-minutes/week, mean (SD)	2582.4 (2251.0)	2158.3 (2262.4)	0.460‡
**Anthropometrics**			
BMI (kg/m^2), mean (SD)	27 (5.5)	27.8 (4.3)	0.562‡
Waist circumference, mean (SD)	96.9 (9.4)	101.9 (10.8)	0.061‡
Hip circumference, mean (SD)	104.4 (7.9)	104.2 (8.9)	0.910‡
**Biochemical profile**			
Uric acid (mg/dl), mean (SD)	5.19 (1.04)	5.3 (0.87)	0.695‡
Urea (mg/dl), mean (SD)	37.7 (12.2)	33.1 (9.5)	0.104‡
Creatinine (mg/dl), mean (SD)	0.85 (0.15)	0.85 (0.16)	0.963‡
Na (mmol/L), mean (SD)	141.3 (1.5)	141.5 (2.1)	0.662‡
K (mmol/L), mean (SD)	4.36 (0.42)	4.45 (0.37)	0.453‡
AST (IU/L), mean (SD)	20.8 (6.1)	20.8 (6.1)	0.970‡
ALT (IU/L), mean (SD)	35.3 (12.3)	35.6 (14.3)	0.934‡
γ–GT (IU/L), mean (SD)	36.7 (19.2)	35.3 (16.7)	0.806‡
Albumin (g/dl), mean (SD)	4.16 (0.36)	4.24 (0.28)	0.421‡
HbA1c (%), mean (SD)	5.69 (0.51)	5.66 (0.32)	0.85‡
Glucose (mg/dl), mean (SD)	93.8 (12.8)	96.7 (14)	0.422‡

Cholesterol (mg/dl), mean (SD)	208 (39.8)	202.9 (43.3)	0.664‡
HDL (mg/dl), mean (SD)	59.9 (16.2)	55 (13.7)	0.258‡
LDL (mg/dl), mean (SD)	116.5 (32)	130.7 (36.3)	0.162‡
Triglycerides (mg/dl), mean (SD)	153.1 (150)	93.7 (37.9)	0.083‡
Atherogenic index, mean (SD)	3.84 (1.48)	3.89 (0.86)	0.876‡
CRP (mg/L), mean (SD)	2.6 (2.67)	2.24 (1.54)	0.585‡

+Pearson's chi-square test; ++Fisher's exact test; ‡Student's t-test

Table S2 MedDietScore, antioxidant food consumption frequency and macronutrient intake by study group. The results are given as N (%) of the total number.

		Group				
		Placebo		Antioxidant		***P***
		**N**	**%**	**N**	**%**	
**MedDietScore, mean (SD)**		31.0 (3.6)		31.2 (5.6)		0.825+
**Energy (kcal), mean (SD)**		1500.5 (534.4)		1637.1 (646.3)		0.370+
**Proteins (gr), mean (SD)**		64.9 (27.1)		64.7 (32)		0.987+
**Carbohydrates (gr), mean (SD)**		111.4 (66.1)		146.9 (89.6)		0.082+
**Fat (gr), mean (SD)**		75.3 (34.6)		80.9 (28.5)		0.484+
**Coffee consumption**	Never/Rarely	3	10.3	6	17.6	0.545++
	1-3 times/month	4	13.8	1	2.9	
	1-2 times/week	3	10.3	3	8.8	
	3-6 times/week	1	3.4	4	11.8	
	Once/day	5	17.2	6	17.6	
	>=twice/day	13	44.8	14	41.2	
**Consumption of herbal beverages**	Never/Rarely	8	27.6	17	50.0	0.329++
	1-3 times/month	7	24.1	3	8.8	
	1-2 times/week	3	10.3	3	8.8	
	3-6 times/week	5	17.2	5	14.7	
	Once/day	6	20.7	5	14.7	
	>=twice/day	0	0,0	1	2.9	
**Consumption of chocolate**	Never/Rarely	8	27.6	11	32.4	0.683++
	1-3 times/month	5	17.2	10	29.4	
	1-2 times/week	12	41.4	10	29.4	
	3-6 times/week	2	6.9	2	5.9	
	Once/day	2	6.9	1	2.9	
	>=twice/day	0	0	0	0	
**Consumption of wine**	Never/Rarely	10	34.5	6	17.6	0.036++
	1-3 times/month	9	31.0	9	26.5	
	1-2 times/week	4	13.8	13	38.2	
	3-6 times/week	1	3.4	3	8.8	
	Once/day	1	3.4	3	8.8	
	>=twice/day	4	13.8	0	0	

+Student's t-test ++Fisher's exact test

Table S3 Anthropometrics at baseline and at follow up. Values are expressed as the mean ± SD.

		Pre	Post	Change	P^1	P^2
		Mean (SD)	Mean (SD)	Mean (SD)		
**Weight (kg)**	Placebo	77 (12.8)	74.7 (14.7)	-2.3 (11.7)	0.098	0.120
	Antioxidant	83 (14.2)	84.1 (14.3)	1.1 (2.2)	0.632	
**BMI (kg/m²)**	Placebo	27 (5.5)	26.2 (4.6)	-0.8 (5)	0.083	0.125
	Antioxidant	27.8 (4.3)	28 (4.2)	0.2 (0.7)	0.724	
**Waist circumference (cm)**	Placebo	96.9 (9.4)	97.7 (10.1)	0.8 (2.9)	0.471	0.722
	Antioxidant	101.9 (10.8)	103 (10.6)	1.1 (3.4)	0.800	
**Hip circumference (cm)**	Placebo	104.4 (7.9)	104.6 (8.5)	0.2 (1)	0.726	0.140
	Antioxidant	104.2 (8.9)	104.6 (9)	0.4 (1.2)	0.092	

[1]*p*-value for the time effect [2]*p*-value from repeated measurements ANOVA. The effects reported include differences between the groups in the degree of change.

Table S4 Biochemical parameters at baseline and at follow up.

	Group	Pre		Post		Change		
		Mean (SD)	Median (IQR)	Mean (SD)	Median (IQR)	Mean (SD)	*P*[1]	*P*[2]
**Hematocrit(%)**	Placebo	42.1 (3.3)	43.2 (40.1;44.1)	41.3 (4.3)	42 (39.2; 44.2)	-0,8 (4.3)	0.924	0.369
	Antioxidant	43.7 (3.4)	44.5 (42.7;45.7)	42.7 (3.4)	43.6 (41; 45.3)	-1,1 (2.5)	0.124	
**Cholesterol (mg/dl)**	Placebo	208 (39.8)	208 (177;238)	221.1(20.3)	216.5(206.5;234)	13.1(20.5)	0.737	0.577
	Antioxidant	202.9(43.3)	190 (177.5;240)	214.1(41.2)	212 (175; 246)	11.2(24.5)	0.179	
**HDL (mg/dl)**	Placebo	59.9 (16.2)	57 (46; 74)	55 (13.2)	53 (45.5; 65.5)	-4.9 (10.4)	0.687	0.178
	Antioxidant	55 (13.7)	53 (48; 58)	56.5 (15.4)	58 (45; 65)	1.5 (7)	0.097	
**LDL (mg/dl)**	Placebo	116.5 (32)	114 (92; 143)	140.4(20.4)	146 (120; 151.5)	23.9(19.7)	0.059	0.849
	Antioxidant	130.7(36.3)	117 (108; 159)	143 (35.2)	142 (120; 178)	12.3(23.3)	0.026	
**Triglycerides (mg/dl)**	Placebo	153.1 (150)	93 (75; 166)	129.4 (52)	118.5 (92; 156)	-23.8(28.7)	0.083	0.205
	Antioxidant	93.7 (37.9)	95 (66; 119)	89 (34.4)	89 (67; 100)	-4.7 (40.4)	0.835	
**Atherogenic index**	Placebo	3.84 (1.48)	3.4 (2.9; 4.4)	4.25 (1.24)	4 (3.4; 4.75)	0.41(1.58)	0.637	0.123
	Antioxidant	3.89 (0.86)	3.6 (3.3; 4.4)	4.09 (1.27)	3.7 (3.2; 4.8)	0.2 (0.7)	0.057	
**Glucose (mg/dl)**	Placebo	93.8 (12.8)	89 (87; 102)	96.4 (17.2)	89.5 (87; 106)	2.6 (10.5)	0.087	0.153
	Antioxidant	96.7 (14)	94 (87; 102)	90.8 (10.3)	87 (84; 98)	-5.9 (8.8)	0.915	
**CRP (mg/L)**	Placebo	2.6 (2.67)	1.6 (1.1; 2.9)	4.85 (4.1)	3.45 (2.2; 6.9)	2.3 (2.4)	0.970	0.710
	Antioxidant	2.24 (1.54)	1.75 (1.1; 3.05)	2.71 (2.12)	2.2 (1.4; 3.4)	0.5 (3.1)	0.510	

[1]*p*-value for the time effect (using logarithmic transformations); [2]*p*-value from repeated measurements ANOVA. The effects reported include differences between the groups in the degree of change (using logarithmic transformations).

Table S5 Macro- and micronutrient intake at baseline and at follow-up.

	Group	Pre		Post		Change	P^1	P^2
		Mean (SD)	Median (IQR)	Mean (SD)	Median (IQR)	Mean (SD)		
**Energy (kcal)**	Placebo	1500.5 (534.4)	1371.8 (1086;1678)	1488.8 (480.9)	1382.6 (1200; 1567)	-11.7 (231.8)	0.622	0.123
	Antioxidant	1637.1 (646.3)	1388.9 (1172; 1910)	1488.4 (380.7)	1484.3 (1144; 1741)	-148.6(595.8)	0.077	
**Proteins (gr)**	Placebo	64.9 (27.1)	58.8 (46,5; 80)	64.8 (26.9)	57.8 (50; 80.2)	-0.1 (10.4)	0.852	0.168
	Antioxidant	64.7 (32)	53.6 (45; 70)	55.9 (20.9)	51.9 (42.3; 67.9)	-8.9 (34.7)	0.063	
**Carbohydrates (gr)**	Placebo	111.4 (66.1)	100 (64.7; 125.2)	122.3 (69.3)	110.3 (83.4; 134)	10.9 (31.7)	0.401	0.099
	Antioxidant	146.9 (89.6)	114.4 (94.6; 57.1)	128.8 (58.4)	120 (91.4; 149.3)	-18.1 (75.5)	0.121	
**Fat (gr)**	Placebo	75.3 (34.6)	69.1 (60; 77.5)	70.2 (31.1)	67 (50; 75.6)	-5.1 (11.2)	0.975	0.535
	Antioxidant	80.9 (28.5)	75.3 (65; 100)	78.1 (25.6)	80 (60; 94.1)	-2.8 (24.9)	0.368	
**b-carotene (μg)**	Placebo	2082.2 (3341.2)	269.1 (68.5; 3615)	1892.4 (2838.9)	590.3 (125.1;2610.9)	-189.8(1130.5)	0.074	0.247
	Antioxidant	1238.2 (2404)	149.9 (75.8; 759.9)	2458.4 (8054.4)	265.4 (74.5; 1180.3)	1220.3(7810.3)	0.729	
**Vitamin A (μg)**	Placebo	329 (326.9)	212.4 (124.8; 460.6)	305.4 (229.7)	242.9 (140.6;366)	-23.6 (228.4)	0.177	0.252
	Antioxidant	257.9 (229.5)	192.7 (118.5;281.4)	444.7 (868.3)	172.6 (90.1;337.5)	186.8 (813.6)	0.860	
**Vitamin C (mg)**	Placebo	66.8 (81.5)	36.6 (15.7;89.6)	79.7 (105.7)	36.6 (13.6;111.9)	12.9 (52.9)	0.485	0.297
	Antioxidant	60.9 (58.7)	40.7 (17.8;84.8)	60.6 (69.3)	31.6 (18;78)	-0.3 (54.9)	0.427	
**Selenium (μg)**	Placebo	59.4 (42.1)	47.2 (33.4;86.1)	70.8 (44.7)	67.5 (32.8;100.5)	11.4 (25.4)	0.084	0.180
	Antioxidant	71.1 (62.8)	57.4 (24.3;105.5)	58.1 (40)	52.5 (24.4;88.6)	-13.1 (64.3)	0.997	
**Zinc (mg)**	Placebo	7.33 (3.12)	6.32 (5.22;9.32)	7.4 (3.94)	6.46 (3.93;10.4)	0.07 (2.97)	0.606	0.552
	Antioxidant	6.53 (3.34)	6.27 (5;8.17)	6.79 (3.64)	5.99 (3.88;9.13)	0.26 (3.83)	0.755	
**cystine (mg)**	Placebo	391.1 (310.7)	258.7 (201.4;583.9)	401.8 (302.1)	345.4 (166;519.9)	10.7 (239)	0.465	0.536
	Antioxidant	442.3 (361.5)	249.8 (142.4;824.6)	404.7 (333.3)	289 (146.6; 588.8)	-37.5 (333.6)	0.926	

[1]*p*-value for the time effect (using logarithmic transformations); [2]*p*-value from repeated measurements ANOVA. The effects reported include differences between the groups in the degree of change (using logarithmic transformations)
